# Comparative Transcriptomics of IBD Patients Indicates Induction of Type 2 Immunity Irrespective of the Disease Ideotype

**DOI:** 10.3389/fmed.2021.664045

**Published:** 2021-05-31

**Authors:** Miguel Gonzalez Acera, Jay V. Patankar, Leonard Diemand, Britta Siegmund, Markus F. Neurath, Stefan Wirtz, Christoph Becker

**Affiliations:** ^1^Department of Medicine 1, University of Erlangen-Nuremberg, Erlangen, Germany; ^2^Deutsches Zentrum Immuntherapie (DZI), Erlangen, Germany; ^3^Charité—Universitätsmedizin Berlin, Corporate Member of Freie Universität Berlin, Humboldt-Universität zu Berlin and Berlin Institute of Health, Medizinische Klinik für Gastroenterologie, Infektiologie und Rheumatologie, Berlin, Germany

**Keywords:** IBD, type 2 immunity, intestine, chronic inflammation, ulcerative colitis

## Abstract

Inflammatory cytokines initiate and sustain the perpetuation of processes leading to chronic inflammatory conditions such as inflammatory bowel diseases (IBD). The nature of the trigger causing an inflammatory reaction decides whether type 1, type 17, or type 2 immune responses, typically characterized by the respective T- helper cell subsets, come into effect. In the intestine, Type 2 responses have been linked with mucosal healing and resolution upon an immune challenge involving parasitic infections. However, type 2 cytokines are frequently elevated in certain types of IBD in particular ulcerative colitis (UC) leading to the assumption that Th2 cells might critically support the pathogenesis of UC raising the question of whether such elevated type 2 responses in IBD are beneficial or detrimental. In line with this, previous studies showed that suppression of IL-13 and other type 2 related molecules in murine models could improve the outcomes of intestinal inflammation. However, therapeutic attempts of neutralizing IL-13 in ulcerative colitis patients have yielded no benefits. Thus, a better understanding of the role of type 2 cytokines in regulating intestinal inflammation is required. Here, we took a comparative transcriptomic approach to address how Th2 responses evolve in different mouse models of colitis and human IBD datasets. Our data show that type 2 immune-related transcripts are induced in the inflamed gut of IBD patients in both Crohn's disease and UC and across widely used mouse models of IBD. Collectively our data implicate that the presence of a type 2 signature rather defines a distinct state of intestinal inflammation than a disease-specific pathomechanism.

## Introduction

Immune responses that detect and eliminate multicellular metazoan parasites and allergic reactions have evolved fundamentally differently than those that tackle other immune challenges. The tissue destruction caused by such parasitic infections and allergic reactions, signals an “eliminate and repair” type of innate immune response, characterized by the emergence of the type 2 helper T cells (Th2) (1). The “type 2” nomenclature was initially coined after Th2 lymphocytes, thought of as being the sole cellular sources capable of producing the core type 2 cytokines interleukin (IL)-4, IL-5, and IL-13 (2). However, key discoveries in the field of innate immunity revealed the presence of new cellular sources of type 2 cytokines (3). For e.g., the type 2 innate lymphoid cells, which also express the polarizing transcription factor GATA3 are rich sources of IL-4 and express IL-5 and IL-13 (4). The effector cells of type 2 responses are epithelia, myeloid cells and granulocytes including dendritic cells, macrophages, neutrophils, basophils, eosinophils, and mast cells and the various secretory and absorptive lineages of epithelial cells. Multiple, differentially polarized subsets of T cells are involved in disease pathogenesis in inflammatory conditions, for e.g., Th1, Th2, Th9, and Th17 (5, 6).

It has been speculated that a breakdown in type 2-mediated processes is the underlying cause of the failure of tissue repair and healing in chronic debilitating conditions affecting mucosal tissues (1). However, our understanding of whether this hypothesis holds true is limited. Studies on chronic diseases affecting the gut such as inflammatory bowel diseases (IBD) show a dichotomous relationship between type 2 responses and the two predominant types of IBD, namely Crohn's disease (CD) and ulcerative colitis (UC). Both CD and UC are chronic mucosal inflammatory diseases, an elevated expression of type 2 cytokines may be detected in the tissues and serum of UC, but not of CD patients, however, this concept has recently been challenged (7, 8). Therefore, elevation in type 2 cytokines does not establish a causal connection between UC and type 2 immune responses and crucial questions on when and why such an elevation may be observed remain poorly addressed. Administration of neutralizing antibodies against IL-13 failed to induce any clinical response in UC patients, which begs the question whether defective signaling via respective receptors could cause a compensatory elevation in the expression of the type 2 cytokines in UC (9–11). Indeed some of the well-documented downstream effects of type 2 cytokines such as increased smooth muscle proliferation and cholinergic activation, and elevated mucin production are not uniquely seen to occur in UC, indicating that there is, at least, a partial functional blockade in the mediation of type 2 responses despite the elevation in their levels. By contrast, some studies indicate that elevated type 2 responses in CD patients during parasite infections can in fact prove therapeutically beneficial (12). It is therefore complicated to dissect how type 2 cytokines influence IBD.

Strong type 2 responses are elicited in mucosal tissues during allergic conditions or helminth infections. Therefore, the most common mouse models to study type 2 immune responses are based on infecting mice with parasitic helminths. Some of the most common nematode worms used to provoke mucosal type 2 responses in mice are *Nippostrongylus brasiliensis* and *Heligmosomoides polygyrus* (13). Another mouse model, which is reported to show a predominant type 2 response, is the oxazolone colitis model (14, 15). Other common mouse models of colitis such as the chemically induced dextran sulfate sodium (DSS) colitis and tri-nitrobenzidium sulfate (TNBS) colitis are described to have a more mixed Th 1, Th 17 response (16). Whereas, the chronic DSS colitis model is described to involve more of a type 2 immune response (16). One of the most commonly employed models to study the contribution of T cells to the pathogenicity of colitis is the transfer colitis mouse model, which is more of a type 1 model (17). Although these generalized distinctions hold out, there has also been evidence that indicates the contrary. Therefore, the dogmatic view of UC as a predominantly type 2 and CD a type 1 immune disease has been challenged. This is important given that both therapeutic and diagnostic attempts in IBD have evolved around our understanding of the immune homeostasis.

In order to improve our understanding of the type 2 response in IBD, we present here a comparative transcriptomic analysis of various mouse models and human cohorts of IBD using a well-defined list of markers of type 2 immune responses. Our data show that an elevation in type 2 responses is a generalized phenomenon seen intestinal inflammation and identifies a core network of type 2 associated genes that is shared between preclinical models and patients of IBD.

## Materials and Methods

### Mouse Models of Colitis

The C57Bl6 strain has been widely used in colitis research and was purchased from Charles river laboratories GmbH (Sulzfeld, Germany). All mice included in this study were of both genders and aged between 8 and 18 weeks. Acute and chronic DSS, Oxazolone, and T-cell transfer colitis were induced as previously described (18, 19). At the end of the colitis induction period, animals were euthanized and tissues were harvested for further analysis. The housing, animal care and experimentation was performed according to the institutional guidelines that were preapproved by the ethics commission of Lower Franconia and Rhineland.

### RNA Extraction and mRNA Sequencing

The RNA from the tissue samples were harvested using the peqGold total tissue RNA kit (peqlab GmbH) according to the manufacturer's instructions. The RNA degradation, contamination and quantification were checked using a combination of 1% agarose gels checked using the NanoPhotometer® spectrophotometer (IMPLEN, CA, USA), the Nanodrop (thermofischer), the Qbit (Thermo) and the RNA Nano 6000 Assay Kit of the Bioanalyzer 2100 system (Agilent Technologies, CA, USA).

For library preparation for transcriptome, sequencing a total amount of 1 μg RNA per sample was used as input material per sample. Sequencing libraries were generated using NEBNext® UltraTM RNA Library Prep Kit for Illumina® (NEB, USA) following manufacturer's recommendations and index codes were added to attribute sequences to each sample. Briefly, mRNA was purified from total RNA using poly-T oligo-attached magnetic beads. Fragmentation was carried out using divalent cations under elevated temperature in NEBNext First Strand Synthesis Reaction Buffer (5X). First strand cDNA was synthesized using random hexamer primer and M-MuLV Reverse Transcriptase (RNase H-). Second strand cDNA synthesis was subsequently performed using DNA Polymerase I and RNase H. Remaining overhangs were converted into blunt ends via exonuclease/polymerase activities. After adenylation of 3' ends of DNA fragments, NEBNext Adaptor with hairpin loop structure were ligated to prepare for hybridization. In order to select cDNA fragments of preferentially 150–200 bp in length, the library fragments were purified with AMPure XP system (Beckman Coulter, Beverly, USA). Then 3 μl USER Enzyme (NEB, USA) was used with size-selected, adaptorligated cDNA at 37°C for 15 min followed by 5 min at 95°C before PCR. Then PCR was performed with Phusion High-Fidelity DNA polymerase, Universal PCR primers and Index (X) Primer. At last, PCR products were purified (AMPure XP system) and library quality was assessed on the Agilent Bioanalyzer 2100 system.

The clustering of the index-coded samples was performed on a cBot Cluster Generation System using PE Cluster Kit cBot-HS (Illumina) according to the manufacturer's instructions. After cluster generation, the library preparations were sequenced on an Illumina platform and paired-end reads were generated. Raw data (raw reads) of FASTQ format were firstly processed through in-house scripts. In this step, clean data (clean reads) were obtained by removing reads containing adapter and poly-N sequences and reads with low quality from raw data. At the same time, Q20, Q30, and GC content of the clean data were calculated. All the downstream analyses were based on the clean data with high quality. Reference genome and gene model annotation files were downloaded from NCBI, UCSC and Ensembl directly. Paired-end clean reads were mapped to the reference mouse genome (GRCm38.p6) using STAR software (20) (2.7.0d).

### Meta-Analyses of Publicly Available Datasets

#### Mouse Models for Helminth Infection

Transcriptomic data from the duodenal samples of mice infected with the parasite *H. polygyrus* that has been previously published was obtained from NCBI's Gene Expression Omnibus (GEO) https://www.ncbi.nlm.nih.gov/gds (GSE102789). Raw RNAseq data was mapped against the mouse genome and processed and analyzed using the same procedures and tools described above for the mouse colitis models.

#### Human Inflammatory Bowel Disease Cohorts

Transcriptomic data from the respective intestinal tissues from IBD patients from the previously published studies were obtained from –

a) NCBI's Gene Expression Omnibus (GEO) https://www.ncbi.nlm.nih.gov/gds and from
b) EBI's Array Express https://www.ebi.ac.uk/arrayexpress/.

The specifics of the cohorts and the respective accession numbers are provided here:

**Table:** 

**Cohort**	**Accession**	**No. of Samples**	**Tissue**	**References**
WashU	E-MTAB-5783	36 CD, 32 Control	Ileum	(21)
RISK_I	GSE57945	202 CD, 60 UC, 39 Control	Ileum	(22)
PSC	E-MTAB-7915	10 UC, 10 PSC, 10 Control	Colon	(23)
PROTECT	GSE109142	206 UC, 20 Control	Rectum	(24)
RISK_R	GSE117993	92 CD, 43 UC, 55 Control	Rectum	(24)

*CD, Crohn's disease; UC, ulcerative colitis; PSC, primary sclerosing cholangitis*.

Where applicable, microarray data and its annotation was downloaded and processed using the limma R package [3.42.2] (25). Raw RNAseq data was mapped against the human genome [GRCh38.p13], processed and analyzed using the same procedures and tools described above for the mouse colitis models.

Network extension for detection of pathways and physical interactors for a subset type 2 identifier genes that were commonly regulated between mouse and human samples was determined using the freely available tool Genemania for Cytoscape 3.7 (26, 27).

## Statistical Analysis of Differential Gene Expression

FeatureCounts (v2.0.1) was used to count the read numbers mapped of each gene included in the Ensembl database 21. Then the median of ratios of each gene were calculated, based on the sequencing depth and the RNA composition. Although the Reads Per Kilobase of exon model per Million (RPKM) is currently one of the most commonly used methods for normalization of RNA seq data, the median of ratios used by DESeq2 is more accurate for the differential expression analysis22.

Differential expression analysis between two conditions/groups (three biological replicates per condition) was performed using DESeq2 (v 1.26.0) R package22. DESeq2 provides statistical routines for determining differential expression in digital gene expression data using a model based on the negative binomial distribution22. The resulting *P*-values were adjusted using the Benjamini and Hochberg's approach for controlling the False Discovery Rate (FDR). Genes with an adjusted *P* < 0.05 found by DESeq2 were assigned as differentially expressed.

## Results

### Selection of an Identifier Gene Set Representing Type 2 Responses

To generate a discovery gene set for type 2 immunity that would then be used to query transcriptomic datasets, we surveyed gene sets in the molecular signatures database (MSigDB, https://www.gsea-msigdb.org/gsea/msigdb) (28). The MSigDB database serves as a standard resource for gene sets used in gene set enrichment analyses. A list of all MSigDB gene sets used for this analysis is available as Supplemental Table 1. Surprisingly, an integrated list of the 15 gene sets that we investigated, failed to show the necessary attributes of a canonical type 2 signature. Therefore, based on reliable literature resources, we compiled a list of 39 genes, which are known to be classical markers for type 2 immune responses (Table 1). This list includes genes categorized into five major groups: cytokines, transcription factors, chemokines, receptors, and other genes, which served as a molecular identifier to interrogate transcriptomic datasets and associate a specific type 2 signature (Table 1).

**Table 1:** Identifier gene set for type 2 immunity.

**Symbol**	**Full name**	**Description**	**References**
*Il33*	Interleukin 33	Cytokine that drives production of Th2 associated cytokines. Expressed in a wide range of cells	(29)
*Il25*	Interleukin 25	Cytokine that drives production of Th2 associated cytokines. Expressed in a wide range of cells	(2)
*Il4*	Interleukin 4	Cytokine that induces differentiation of naive Th cells to Th2	(30)
*Il5*	Interleukin 5	Cytokine that stimulates IgA secretion and activates eosinophils	(31)
*Il9*	Interleukin 9	Cytokine that regulates hematopoietic cells activating several STAT signaling pathways, stimulating cell proliferation, and preventing apoptosis	(32, 33)
*Il10*	Interleukin 10	Anti-inflammatory cytokine that down regulates the expression of Th1 cytokines	(34, 35)
*Il13*	Interleukin 13	Cytokine with similar effects in immune cells than those caused by IL-4	(36, 37)
*Il31*	Interleukin 31	Pruritic cytokine expressed by Th2 cells in response to stimulation with type 2 cytokines	(38, 39)
*TLSP*	Thymic stromal lymphoproietin	Cytokine that plays a role on activating Dendritic Cells	(40)
*AREG*	Amphiregulin	Epidermal growth factor secreted by ILC2 after tissue damage.	(41, 42)
*GATA3*	GATA binding protein 3	Transcription factor that stimulates the producion of IL-4, IL-5 and IL-13.	(43)
*MAF*	Avian musculoaponeurotic fibrosarcoma oncogene	Transcription factor that regulates the expression of IL-4 and attenuates type 1 response.	(44)
*STAT6*	Signal transducer and activator of transcription 6	Transcription factor that acts as the intracellular effector of IL-4 in Th2 cells	(45)
*CCL1*	Chemokine (C-C motif) ligand 1	Chemokine that acts as a chemoattractant for multiple immune cells.	(46, 47)
*CCL8*	Chemokine (C-C motif) ligand 8	Chemokine that acts as a chemoattractant for multiple immune cells.	(48)
*CCL17*	Chemokine (C-C motif) ligand 17	Chemokine that induces chemotaxis in T cells.	(49)
*CCL22*	Chemokine (C-C motif) ligand 22	Chemokine that induces chemotaxis in T cells.	(49)
*IL1RL1*	Interleukin 1 Receptor like protein 1	Receptor for IL-33	(50)
*PTGDR2*	Prostaglandin D2 receptor 2, Interleukin 52 (CRTH2)	Prostaglandin receptor that mediates in the chemotaxis of Th2 cells	(51)
*IL17RB*	Interleukin 17 Receptor B	Receptor for IL25	(52)
*CRLF2*	Cytokine receptor-like factor 2	Receptor for TSLP	(53)
*CCR4*	C-C chemokine receptor type 4	Receptor for multiple chemokines, including CCL17 and CCL22.	(54)
*CCR8*	C-C chemokine receptor 8	Receptor for CCL1	(55)
*IL4R*	Interleukin 4 Receptor subunit alpha	Receptor for IL-4 and IL-13, forms a complex with IL-13RA1	(56)
*IL13RA1*	Interleukin 13 Receptor alpha 1	Receptor for IL-4 and IL-13, forms a complex with IL-4R	(57)
*IL31RA*	Interleukin 31 receptor A	Receptor for IL-31	(58)
*IL9R*	Interleukin 9 Receptor	Receptor for IL-9	(59)
*DENND1B*	DENN domain-containing protein 1A	Protein containing a DENN domain, which interacts with Rab family GTPases	(60)
*ITK*	IL-2-inducible T-cell kinase	Tyrosine kinase that plays a role on the differentiation and proliferation of Th2 cells	(61)
*ARG1*	Arginase 1	Metabolic enzyme and marker of activated ILCs	(62)
*ARG2*	Arginase 2	Paralog of ARG1 and marker of activated T cells	(63)
*ECM1*	Extracellular matrix protein 1	Extracellular protein that contributes in the manteinance of the epithelium	(64)
*PRKCZ*	Protein kinase C zeta	Protein kinase C that regulates differentiation of T cells	(65)
*RETNLA*	Resistin like molecule alpha (FIZZ)	Molecule increased in inflammatory and allergic responses	(66)
*RETNLB*	Resistin like molecule beta	Molecule increased in inflammatory and allergic responses	(67)
*CHIL3*	Chitinase-like protein 3	Pseudo chitinase expressed in IECs and macrophages in inflammation	(68)
*MUC5AC*	Mucin 5AC	Gene involved in the production of mucus	(69)
*MRC1*	Mannose receptor C-type 1	Protein expressed by intestinal macrophages	(70)

The genes included in the cytokines group are involved in multiple types of signaling for the activation of the type 2 response. These include the classical type 2 cytokines *Il4, Il5, IL9*, and *IL13* that are involved in the activation of the effector cells (30–33, 36, 37). The cytokines *Il33, Il25*, and *TLSP* are primarily produced by parenchymal cells in response to damage, thereby rapidly activating type 2 ILCs (ILC2s) and other immune cells in an early activation stage (2, 29, 40). The ILC2s and Treg cells secrete AREG in order to help repair the damaged epithelium (41, 42). Finally, IL10 is produced by macrophages and lymphocytes, and has an anti-inflammatory function, in order to maintain intestinal homeostasis (34, 35).

Among the key transcription factors (TF) that regulates type 2 polarization and expression of multiple cytokines is *GATA3* (43). Another key regulatory TF assigned to type 2 responses is MAF, which controls the production of *Il4*, and attenuates type 1 signaling (44). The *IL4* signaling is crucial in dictating type 2 responses and its actions are mediated by the activation of the TF *STAT6*, which regulates a plethora of type 2 related effects including polarization and recruitment of Th2 cells (45).

Among the chemokines that represent the type 2 signature were *CCL1* and *CCL8* that promote migration and activation of ILC2s and Treg cells. *CCL17* and *CCL22* have similar effects, dendritic cells (DC) produce both and they interact with T helper cells (46–49).

Key cytokine receptors, which influence the type 2 polarization and function are *IL4R1* (lymphocytes, ILC, fibroblasts, and epithelium), *IL9R* (eosinophils, mast cells, ILC2s), *IL13RA1* (ILC, granulocytes, epithelial cells, fibroblasts), and *IL31RA* (monocytes, epithelium) (56, 57, 59). Other cytokine receptors which influence type 2 behaviors include the IL-33 receptor subunit *IL1RL1* (hematopoietic), which has been seen involved in allergic responses and *IL17RB* (ILC2s, monocyte, Tuft cells), which is receptor of *IL25* (50, 52). The receptors for type 2-related chemokines are also included in this group, with the genes *CCR4* (Th2) and *CCR8* (ILCs, T cells) (54, 55). *PTGDR2* is a prostaglandin D receptor expressed in Th2 cells, mediating allergic responses (51). The receptor of *TSLP, CRLF2* (DC, hematopoietic) is also a key component determining the activation and initiation of Th2 responses (40, 53).

Finally, we identified 12 additional genes that are known to control type 2 responses, but do not fall into any of the above categories. Among these were *DENND1B* which is involved in the down-modulation of the T cell receptor, and its absence, malfunction or delay has been associated with asthma and allergic response (60). The Tec family tyrosine kinase *ITK*, also included in this group, is required for the production of type 2 cytokines and the differentiation of ILC2s and T cells (61). *ARG1* and *ARG2* are paralogues, both encoding for the metabolic enzyme arginase, which metabolizes L-arginine, and has been identified as a marker of ILC2s and alternatively activated macrophages; and an upstream regulator of these metabolic genes is *ECM1* (62–64). *PRKCZ* encodes an atypical protein kinase C, which is involved in immune surveillance (65). Next, we included *RETNLA* and *RETNLB* that encode proteins of the Resistin family that are generally elevated upon type 2 immune activation via the actions of IL-13 (66, 67). During nematode driven intestinal type 2 responses, the upregulation of the chitinase *CHIL3* is detectable (68). This enzyme controls the degradation of chitin, and is produced and released by intestinal epithelial cells promoting host cell survival and proliferation. Various scenarios of type 2 activation in the intestine have shown that *MUC5AC* is induced in the epithelium and is responsible for the elevated mucus production, characteristic of these models (69). MRC1 is a receptor induced by the type 2 cytokine IL4 and can bind high-mannose structures on parasite walls, aiding their neutralization and engulfment (70).

### Transcriptomic Comparison Across Multiple Mouse Models of IBD Reveals a Non-discriminatory Regulation of Type 2 Response

**Figure 1:**
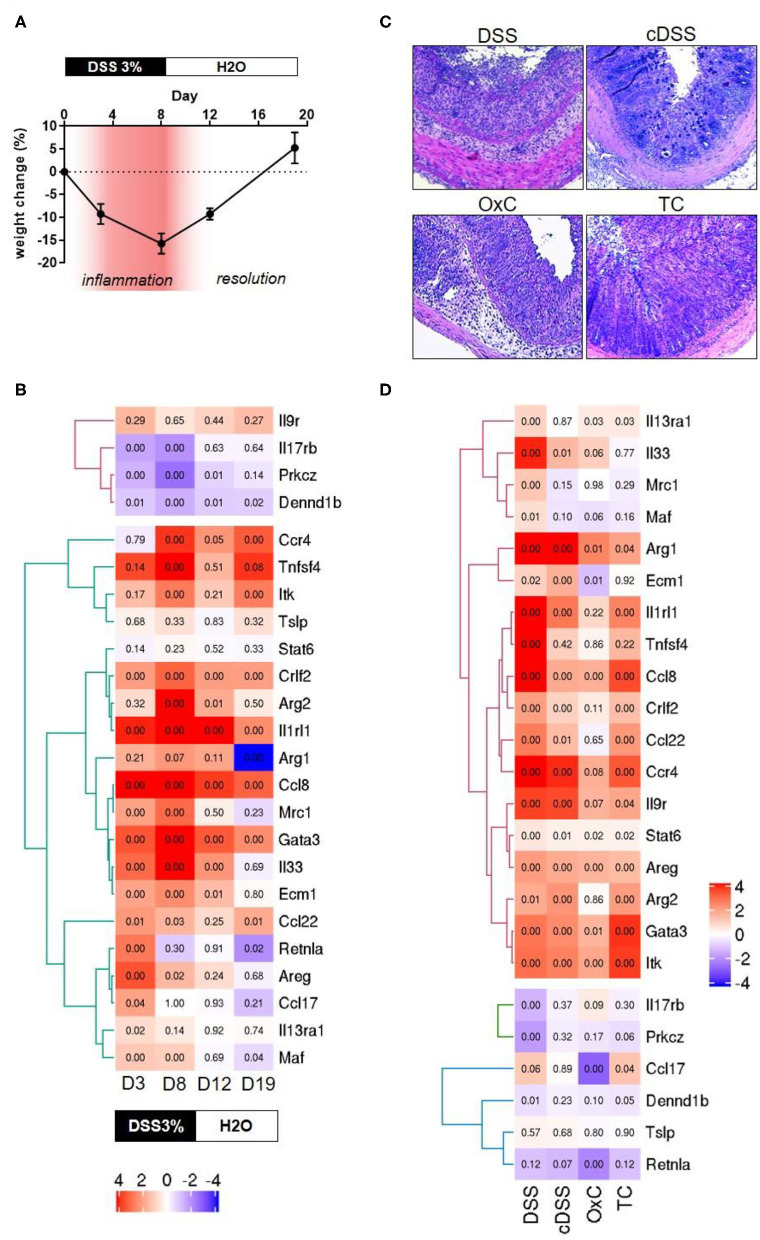
Transcriptomic analyses of type 2 identifiers in multiple mouse models of colitis. **(A)** percent change in body weight from baseline and DSS treatment paradigm. Time points representing inflammation are represented in red **(B)** Heatmap depicting the normalized expression of the indicated genes from four different time-points of dextran sulfate sodium (DSS) induced colitis D3 = day 3, D8 = day 8, D12 = day 12, and D19 = day 19. The text boxes below represent the treatment type and duration. Hierarchical clustering shows clear segregation of two distinct groups of genes. **(C)** Representative photomicrographs of H&E-stained tissue sections from the indicated colitis mouse models. DSS, acute dextran sulfate sodium colitis; cDSS, chronic dextran sulfate sodium colitis; OxC, Oxazolone induced colitis; TC, T-cell adoptive transfer colitis. **(D)** Heatmap showing normalized expression levels of the indicated genes the type of regimen used to induce colitis is depicted below. Numbers in each quadrant represent the *p*-value and the legend denotes the fold changes in expression ratios.

In an initial attempt to find discernable patterns of the involvement of type 2 immune responses in different mouse models of IBD, we screened colonic transcriptomes against the type 2 immune response identifier gene set. The mouse models included for this were a time course of dextran sulfate sodium (DSS) colitis representing mild inflammation at day 3, high inflammation at day 8, moderate recovery at day 12 and full recovery from colitis at day 19 of the experimental protocol, characterized by distinct changes in body weights during and after DSS challenge (Figures 1A,B). Apart from these 4 stages of DSS colitis we also included samples of acute DSS colitis, chronic DSS colitis, Oxazolone colitis, and adoptive T-cell transfer colitis, where inflammation was ascertained by histochemical staining (Figures 1C,D). Colonic transcriptomes from unchallenged Rag1 knockout mice and unchallenged C57BL/6 mice were used as control datasets for the T-cell transfer colitis and the rest of the mouse models, respectively. Our exploration yielded 25 genes representing 64% of the identifier gene set which met all requisite criteria for technical thresholds across all the mouse model datasets (Figures 1C,D). Intriguingly, while a type 2 immune signature was evident throughout the course of DSS-induced colitis, we failed to identify a clear type 2 discriminatory signature in specific mouse models and observed that surprisingly, DSS colitis had the most dynamic Th2 response followed by T-cell transfer colitis and lastly oxazolone colitis (Figure 1D). Oxazolone colitis showed the mildest regulatory shift to a type 2 response with only 5 genes of the identifier gene set being significantly regulated. These included *Itk, Ccl17, Retnla, Areg*, and *Ccl8* (Figure 1D). Interestingly, oxazolone colitis was the only dataset in which *Ccl17* was significantly regulated. There was an overall tendency toward upregulation of most of the transcripts that we assessed barring 3 transcripts, *Dennd1b, Prkcz*, and *Il17rb* which showed an overall downregulation and clustered together across all the datasets that we tested (Figure 1D). The protein products of these three genes functionally cooperate downstream of IL-17 signaling to modulate cellular stress response (71). Among all the genes and across all the datasets, the expression of *Tslp* showed the least significant variation in expression making it the only gene in the identifier that failed to show significant expression changes in at least one of the datasets (Figures 1C,D). The gene that showed high similarity across all the datasets was *Ccl8*, which was significantly upregulated in all the tested datasets (Figures 1C,D).

Several genes such as *Gata3, Itk, Ccl8, Ccl22, Ccr4, Crlf2, Il9r, Il1rl1*, and *Arg2* are regulated concordantly in T-cell transfer colitis, acute DSS colitis and the time point representing high inflammation in the DSS time course (Figures 1C,D). Finally, some genes tended to show a greater proportion of upregulation only in the DSS colitis models. These included *Il33, Ecm1, Arg1* and *Mrc1*, and *Maf* (Figures 1C,D). No gene was exclusively upregulated in the Transfer or Oxazolone colitis models. Thus, our analysis fails to identify a discriminatory type 2 signature in mouse models of IBD, which are known to have distinct immune phenotypes (14–17).

### Publicly Available Transcriptomic Datasets Show a Non-discriminatory Regulation of Type 2 Identifier Gene Set Between Different IBD Subtypes

**Figure 2:**
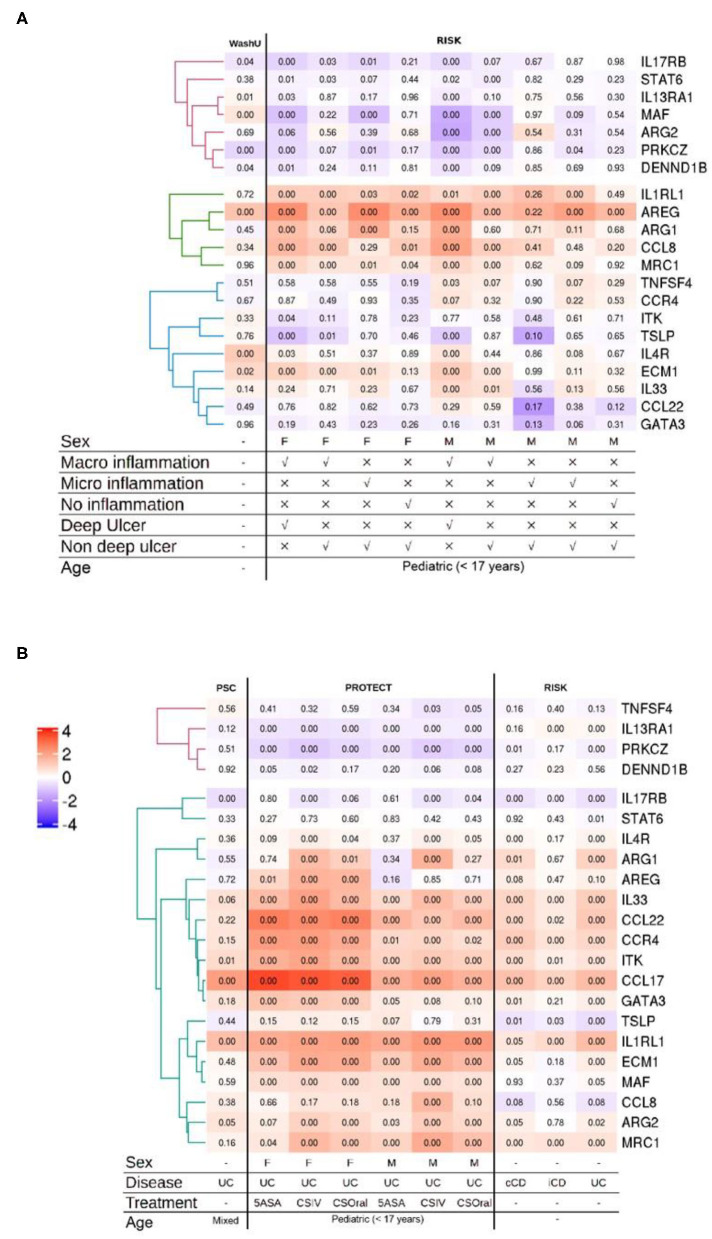
Analysis of multiple IBD cohorts using the type 2 identifier gene set. **(A)** Heatmap of the normalized ileal gene expression profiles from the indicated cohorts of CD patients. Where available, gender, inflammation type and ulceration status are depicted below. **(B)** Heatmap of the normalized gene expression profiles of the indicated genes in the colonic and rectal tissues from IBD patients. Where available, gender, disease diagnosis (UC, Ulcerative Colitis; iCD, ileal Crohn's disease; and cCD, colonic Crohn's disease) and treatments (5-ASA, 5-aminosalicylic acid; CSIV, Cyclosporin intravenous; and CSOral, Cyclosporin oral) are indicated below. Numbers in each quadrant represent the *p*-value and the legend denotes the fold changes in expression ratios.

Classically, UC has been classified as having a dominant type 2 immune signature. This view has always been controversial and there is evidence in support of and against this view (72, 73). To address whether the type 2 gene set identifier may discriminate between human IBD subtypes, we screened five publicly available transcriptomic datasets representing 22 distinct comparisons against respective control samples from intestinal biopsies of human IBD patients. The datasets represented ileum (Figure 2A) as well as the colon and rectum (Figure 2B) at various degrees of inflammation from CD and UC patients of both genders. After applying the appropriate abundance thresholds our analysis yielded 22 genes representing 56% of the identifier gene set that were analyzed across all the cohorts (Figures 2A,B). A comparison of the overall magnitude of the most upregulated genes, showed that the rectal tissues of UC patients, on an average, had 11 out of the 22 genes upregulated (Figure 2B). These included *ARG2, MAF, EVM1, MRC1, CCL22, GATA3, IL33, ITK, CCR4*, and *IL1RL1*. However, their regulatory behavior was non-discriminatory when comparing disease subset or tissue of origin because several of them were also upregulated in CD patients and in inflamed ileal tissues (Figures 2A,B). The least informative genes vis à vis their significance across all the datasets were *TNFSF4, DENND1B, STAT6*, and *TSLP* with significant changes in expression detectable in just 1, 3, 3, and 4 comparisons, respectively (Figures 2A,B). Whereas, genes *IL1RL1, IL33, ECM1*, and *PRKCZ* were the most informative with significance reached in 15, 12, 12, 12, and 12 comparisons, respectively (Figures 2A,B).

In the analysis of the more severe cases of disease across all the cohorts, the gene *IL1RL1* showed the most significant upregulation in all tissues including ileum, colon and rectum (Figures 2A,B). Remarkably, the upregulation in *CCL22* and *GATA3* was restricted to the colonic and rectal samples with no regulation detected in any of the ileal samples irrespective of IBD subtype and gender (Figures 2A,B). Among these, CCL22 afforded the most discriminatory power against ileal tissues, with none of the ileal datasets showing changes in regulation and only the rectal and colonic datasets showing an upregulation. We also detected a discordant expression pattern for two genes *ARG2* and *MAF* between the ileum and the rectum with an overall tendency of being downregulated in the inflamed ilea and upregulated in the inflamed rectum (Figures 2A,B). Among the comparisons involving inflamed vs. control ileal tissues, the genes *CCL8, ARG1* and *AREG* were upregulated in patients of both genders with endoscopic evidence of macroinflammation with deep ulcers (Figures 2A,B).

A generalized trend toward downregulation was observed for a cluster of 3 genes that included *IL13RA1, PRKCZ*, and *DENND1B* (Figures 2A,B). Among this cluster, *IL13RA1* and *PRKCZ* were significantly downregulated across all the cohorts representing rectal tissues, whereas in ileal cohorts, significance was only reached for the most inflamed ileal tissues with evidence of macroinflammation and deep ulceration (Figures 2A,B).

### Comparative Transcriptomics Reveals Conserved Regulation of a Type 2-Associated Module Shared Between Human IBD and Mouse Models of IBD

**Figure 3:**
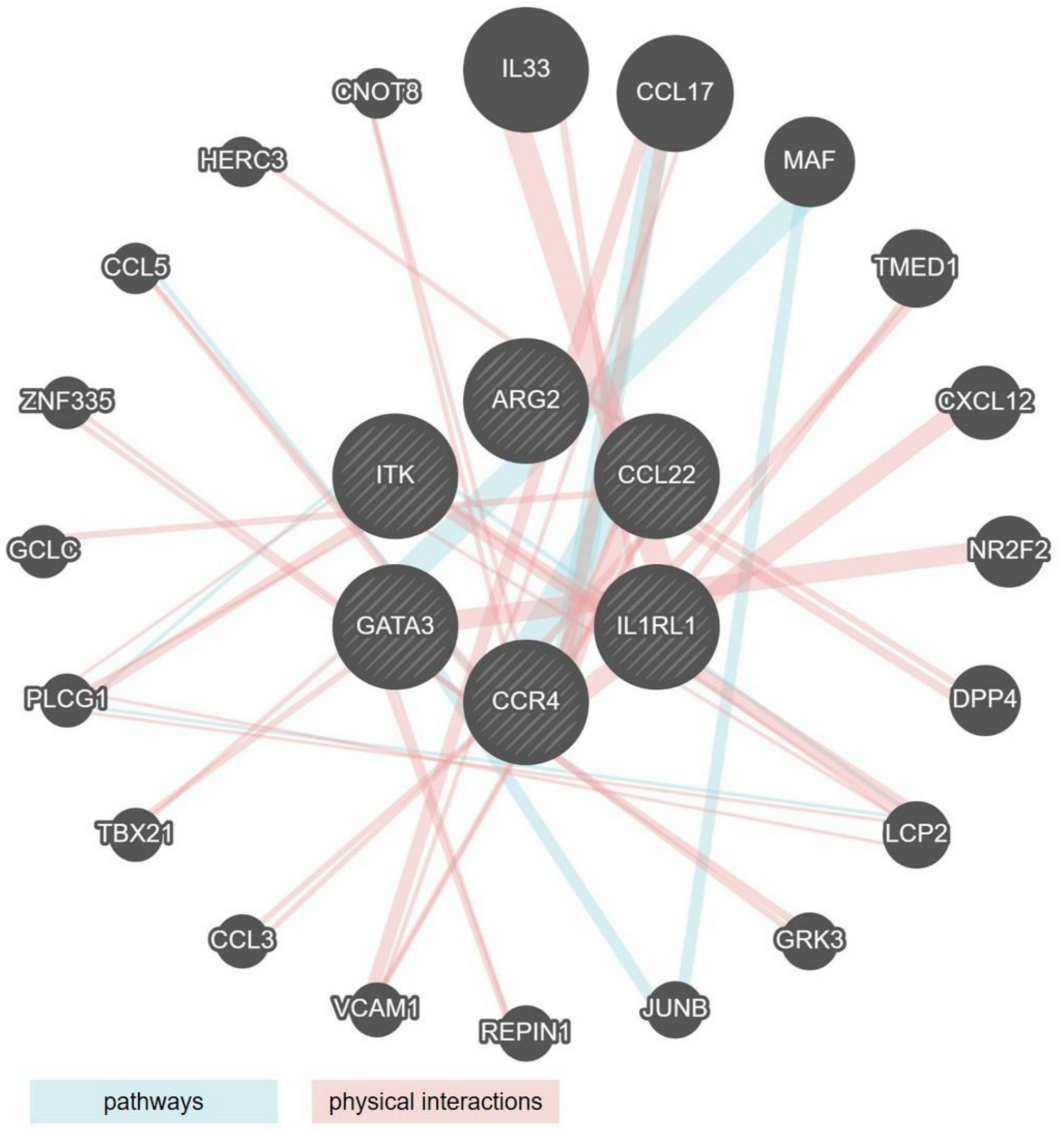
Network analysis of the six type 2 associated genes similarly regulated in human IBD and mouse models of colitis. Radial interaction map of type 2 associated marker genes common to both human IBD and mouse models of colitis (the six central circles, shaded gray). Each non-shaded gray circle represents a predicted pathway interactor (aqua lines) or physical interactor (mauve lines) of the six core factors. Size of each circle represents the rank generated by an automatically selected weighting method in Cytoscape.

By comparing the genes that showed significant regulation (up or down) in the mouse models and the human cohorts, we identified two clusters of genes the regulatory behavior of which was conserved. For both, up as well as down regulated genes that were commonly regulated, the greatest overlap was observed between the mouse models showing acute inflammation and human cohorts that were highly inflamed. The seven genes encoding for *ITK, GATA3, CCL22, IL1RL1, CCR4*, and *ARG2* were upregulated in DSS and T-cell transfer colitis, and in the human colon and rectum cohorts (Figures 1C,D, 2A,B). An analysis of pathways and physical interactors of these genes yielded an extended network (Figure 3). Some of the members of this extended network such as *IL33, CCL17*, and *MAF* were among our type 2 identifier gene set; the others are downstream components, which may participate in regulating type 2 immune response (Figure 3).

Interestingly, and contrary to our expectation, except for *ITK*, the regulatory behavior of none of these seven shared genes was significantly altered in the Oxazolone colitis mouse model, a model previously associated with Th2-driven pathology (Figure 1D). We also identified another module of genes, including *MRC1, ARG2, ECM1, MAF*, and *IL33* that was upregulated in the DSS models as well as in the human rectum cohorts with some degree of overlap with the most inflamed of the ileal cohorts (Figures 1C,D, 2A,B). Among these, the upregulation of only *ARG2* and *MAF* was truly restricted solely to the rectal datasets (Figures 1C,D, 2A,B). Among the DSS mouse models, *Maf* expression was restricted to the mild, high and acute inflammation stages, returned to normal levels during the resolution stage, and remained unaltered in the chronic DSS model (Figures 1C,D).

Interestingly, a cognate behavior for the regulation of *STAT6* and *TSLP* was identified among the mouse models and human cohorts that showed a lack of any significant regulation (Figures 1C,D, 2A,B). The upregulation of *AREG* was observed across all the mouse models except for controls and recovery cohorts and was shared predominantly among in the human ileal but not colonic and rectal ones (Figures 1C,D, 2A,B). Interestingly, the downregulation of the genes *PRKCZ* and *DENND1B* was common among the mouse models and the human cohorts (Figures 1C,D, 2A,B).

## Discussion

Research into the cytokine biology of IBD has nurtured the dogmatic view that different IBD subtypes are characterized by distinct immunotypes (6). This classical view has been repeatedly challenged and recent evidence calls for a revision of this point of view. Although several studies have identified an upregulation in type 2 markers in ulcerative colitis, there is no conclusive evidence that a diagnostic discrimination of UC vs. CD can be attained by measuring the markers of type 1 and type 2 immune responses (8, 74, 75). Thus, the type of mucosal immune response on its own cannot explain the differences in the clinical pathogenesis of CD vs. UC. Preclinical mouse models, which resemble these classical human IBD immunotypes, have been widely employed for gaining a better understanding of disease biology as well as for drug discovery purposes. However, due to a lack of consensus on whether certain archetypal immune responses are associated with a certain mouse model that reflects a specific human IBD subtype, we took advantage of a comparative transcriptomic approach. To our knowledge, such an approach has not been applied so far in gaining an understanding of type 2 immunity in preclinical and clinical IBD.

Classically, colitis induced using the adoptive transfer of CD4^+^CD45RB^high^ T cells into Rag 1, 2 knockout mice that lack T and B cells has been considered to be Th1 -mediated (76). More recently, both Th1 and Th17 cells were broadly implicated in colitogenic disease mechanisms in this model. Another commonly used mouse model for IBD, DSS colitis, is also considered to be predominantly a Th1/Th17 type of colitis, although colitis can develop even in the absence of T cells (77). Conversely, the models of chronic DSS colitis and Oxazolone colitis are considered to be Th2-driven forms of colitis (78). Interestingly, our analysis showed a significant overlap in the expression of cognate type 2 gene set in the DSS as well as the T cell transfer colitis models, which as stated earlier are classically considered type 1-driven models. Interestingly, and contrary to our expectation, chronic DSS colitis and Oxazolone colitis showed the least regulation of the type 2 identifier gene set. Using a single cell sequencing approach, Kiner et al. recently also failed to identify classical T helper cell subsets and could not define distinct Th1, Th17, or Th2 restricted clusters but rather identified phenotypically flexible clusters that depended on the overarching microbial milieu (79). Notably, the lack of Th stereotypes in the gut mucosa was not only observed at steady state, but also when mice were infected with different pathogens including the metazoan parasite *H. polygurus* and *N. brasiliensi* (79). In line with this, we analyzed two transcriptomic datasets, one from the lung and the other from the small intestine of mice that were infected with the helminth parasites *Nippostrongylus brasiliensis* and *Heligmosomoides polygyrus*, respectively (80, 81). Similar to the findings of Kiner et al., we also failed to identify a significant proportion of canonical type 2 response signatures, which were shared between the two tissues.

One technical caveat of transcriptome sequencing methods used in our study is the cutoff for minimum sequence length that guarantees a good sequence read. In our method, this cutoff was 150 base pairs for a paired end sequencing, which inadvertently causes a loss of short length transcripts, which we were unable to include in our analysis. Nonetheless, our analysis contributes to the growing body of evidence that points to a reassessment of the classical immune subtyping in IBD.

In addition, our data reveal a conserved group of type 2 associated genes, which are regulated similarly in the commonly used mouse models of IBD and in human IBD. The analyses presented in our work shows that most of the classical markers of type 2 immune response do not behave in a presumed categorical pattern. We also show that the conception that in preclinical studies of IBD, specific types of immune responses can be modeled using specific mouse models needs to be revised.

## Data Availability Statement

The data for the mRNA sequencing datasets generated in this study for the time course of experimental DSS colitis are available via the array express service of EMBL-EBI through the accession number E-MTAB-9850. All other datasets analyzed in this study along with their accession numbers, references, and sources for the data can be found in the materials and methods section of this article.

## Ethics Statement

The animal study was reviewed and approved by The ethics commission of Lower Franconia and Rhineland.

## Author Contributions

LD: experimentation and analysis. MG, JP, SW, and CB: study concept, design, literature search, experimentation, analysis, interpretation of data, and critical revision of the manuscript. BS: critical revision of the manuscript. All authors contributed to the article and approved the submitted version.

## Conflict of Interest

The authors declare that the research was conducted in the absence of any commercial or financial relationships that could be construed as a potential conflict of interest.
